# A Review of the Proteomic Profiling of African Viperidae and Elapidae Snake Venoms and Their Antivenom Neutralisation

**DOI:** 10.3390/toxins14110723

**Published:** 2022-10-22

**Authors:** Benedict C. Offor, Beric Muller, Lizelle A. Piater

**Affiliations:** 1 Department of Biochemistry, Faculty of Science, University of Johannesburg, Auckland Park 2006, South Africa; 2 South Africa Venom Suppliers CC, Louis Trichardt 0920, South Africa

**Keywords:** antivenom, elapids, proteomics, snake venom, toxins, venomics, viperids

## Abstract

Snakebite envenoming is a neglected tropical disease (NTD) that results from the injection of snake venom of a venomous snake into animals and humans. In Africa (mainly in sub-Saharan Africa), over 100,000 envenomings and over 10,000 deaths per annum from snakebite have been reported. Difficulties in snakebite prevention and antivenom treatment are believed to result from a lack of epidemiological data and underestimated figures on snakebite envenoming-related morbidity and mortality. There are species- and genus-specific variations associated with snake venoms in Africa and across the globe. These variations contribute massively to diverse differences in venom toxicity and pathogenicity that can undermine the efficacy of adopted antivenom therapies used in the treatment of snakebite envenoming. There is a need to profile all snake venom proteins of medically important venomous snakes endemic to Africa. This is anticipated to help in the development of safer and more effective antivenoms for the treatment of snakebite envenoming within the continent. In this review, the proteomes of 34 snake venoms from the most medically important snakes in Africa, namely the Viperidae and Elipdae, were extracted from the literature. The toxin families were grouped into dominant, secondary, minor, and others based on the abundance of the protein families in the venom proteomes. The Viperidae venom proteome was dominated by snake venom metalloproteinases (SVMPs–41%), snake venom serine proteases (SVSPs–16%), and phospholipase A_2_ (PLA_2_–17%) protein families, while three-finger toxins (3FTxs–66%) and PLA_2_s (16%) dominated those of the Elapidae. We further review the neutralisation of these snake venoms by selected antivenoms widely used within the African continent. The profiling of African snake venom proteomes will aid in the development of effective antivenom against snakebite envenoming and, additionally, could possibly reveal therapeutic applications of snake venom proteins.

## 1. Introduction

Snakebite envenoming is a neglected tropical disease (NTD) that causes serious public health issues. Snakebite envenoming mainly affects people from poorly developed and politically marginalised rural tropical and subtropical communities in India, Southeast Asia, sub-Saharan Africa, and Latin America [1,2]. According to the World Health Organization (WHO), snakes bite an estimated 1.2–5.5 million people annually, and as many as 1.8–2.7 million are envenomed, with mortality rates from the envenoming complications of more than 81,000–138,000 deaths and up to 400,000 survivors permanently deformed [1,3,4,5]. One million snakebites have been estimated in Africa (mainly in sub-Saharan Africa) per annum, leading to over 100,000 envenomings and over 10,000 deaths. A study showed that over 5000 to 14,000 amputations occur annually in sub-Saharan Africa [6]. A recent meta-analysis study of the burden of snakebite envenomation-related deaths, amputations, and post-traumatic stress disorder in 41 sub-Saharan African countries is estimated at 1.03 million DALYs (disability-adjusted life years) annually [7].

Lack of epidemiological data and underestimated figures on snakebite envenoming-related morbidity and mortality impedes snakebite prevention and antivenom treatment [6]. The menace of snake envenoming in Africa can be alleviated by an accurate collection of epidemiological data, educating the at-risk population on the need to use health centres, improvement to the accessibility of antivenoms, and adequate training of health personnel [8].

The snake venom toxins cause diverse clinical manifestations ranging from local tissue damage to life-threatening systemic effects, including neurotoxicity (neuromuscular paralysis), haemotoxicity (haemorrhage and coagulopathy), and/or cytotoxicity (swelling, blistering, and tissue necrosis) [1,9,10]. The intra- and interspecies differences in venom protein compositions may be the cause of differences in venom toxicity and pathogenicity, which can undermine the efficacy of antivenom therapies adopted in the treatment of snakebite envenoming [11]. For instance, intra-specific variation was observed in the venom profile and immunogenicity of the African puff adder, *Bitis arietans*, within sub-Saharan Africa [12]. In Africa, elapids and viperids are medically important snakes that cause most of the snakebite envenoming and are the main focus of this review. The families of elapids include cobras (*Naja* spp.) and mambas (*Dendroaspis* spp.), while the viperids include adders (*Bitis* spp.) and saw-scaled/carpet vipers (*Echis* spp.) [13]. Snakebite envenoming by Viperidae mainly induces myotoxicity and haemotoxicity, whereas the Elapidae leads to cytotoxicity, neurotoxicity, and cardiotoxicity [14]. Elapidae-associated neurotoxicity is characterised by the block of nerve-muscular junction [15].

In 2017, following a recommendation by the WHO’s strategic and technical advisory group for NTD, snakebite envenoming was listed as an NTD. In 2018, this recommendation was adopted by the 71st World Health Assembly. Later, in 2019, the WHO provided its 2019–2030 global strategy for the prevention and control of snakebite envenoming aimed at reducing deaths and disabilities by 50% before 2030 [5,16]. The key goals include (i) empowering and engaging communities, (ii) ensuring safe, effective treatment, (iii) strengthening health systems, and (iv) increasing partnerships, coordination, and resources with strong collaboration. To achieve the above target, in 2021, the WHO launched new snakebite information and a data platform encompassing an open-access online platform that allows access to venomous snake geographical locations, ecology, epidemiology, antivenom treatments and distribution stockpile (https://www.who.int/teams/control-of-neglected-tropical-diseases/snakebite-envenoming/snakebite-information-and-data-platform/overview#tab=tab_1 accessed on 29 August 2022). The platform also helps in data sharing, collaboration, awareness and the education of people. Future directives to better understand and eradicate snakebite envenomation in Africa require multidisciplinary approaches alongside preclinical evaluation and production of multi-antivenoms across Africa [17]. Proteome characterisation of snake venoms and large-scale antivenom production, amongst other stated WHO goals, are anticipated to alleviate the menace of snakebite envenomation in Africa. Thus, the analyses of proteomes of venoms of medically important snakes in Africa are reviewed. Additionally, we report the neutralisation and abilities of selected antivenoms in the African market against the venoms.

## 2. Characteristics of Snake Venom Proteins

Snake venom contains several compounds dominated by protein mixtures that include phospholipase A_2_ (PLA_2_s), metalloproteases, serine proteases, L-amino acid oxidases (LAAO), disintegrins (DIS) and C-type lectins (CTL) [18,19]. Three-finger toxins (3FTxs), PLA_2_s, snake venom metalloproteinases (SVMPs), and snake venom serine proteases (SVSPs) encoded by multilocus gene families are the most abundant protein families component of snake venom, which exhibit multifunctionality [9]. These components of snake venom play a key role in snakebite envenomations and have also been adopted in various therapies, including anticancer, antihaemorrhagic and antihypertensive agents [14,20,21,22]. Most of the abundant protein families in Viperidae and Elapidae are elaborated in Table 1.

### 2.1. Snake Venom Phospholipase A_2_s

Snake venom PLA_2_s are enzymes that play major toxic roles in envenomation. These enzymes have been identified in the venoms of all kinds of snake families, including Viperidae and Elapidae [13] (Table 1). These are small proteins with a molecular mass of ~13–15 kDa, which require Ca^2+^ for activities [39]. Most PLA_2_s catalyse the hydrolysis of glycerophospholipids at the *sn*-2 position of the glycerol backbone, releasing lysophospholipids and fatty acids [23]. These hydrolysis products have many downstream physiological roles. Snake venom PLA_2_s induce several pharmacological effects in victims, such as neurotoxicity, cytotoxicity, cardiotoxicity, myotoxicity, oedema, and anticoagulation. These effects are linked to their multiple functional sites and diverse receptors [18]. Most viperids and some elapids venoms cause local tissue damage, such as myonecrosis, primarily through the activities of PLA_2_s that disrupt the integrity of the plasma membrane of muscle fibres [1]. The Viperidae venom PLA_2_ has both monomeric and dimeric enzymatic forms. The monomeric PLA_2_ has cytotoxicity activities, while the dimeric form possesses both cytotoxicity and neurotoxicity activities [39]. Comparatively, in the case of Elapidae, monomeric, dimeric and trimeric PLA_2_ with neurotoxicity activities have been identified [39,40,41]. Several subtypes of PLA_2_ exist, but they are classified into group I and group II based on the disulphide bond. Group II is further classified into Asp49 PLA_2_ and Lys49 PLA_2_ myotoxins involved in cytotoxicity activities in Viperidae venom [42]. PLA_2_ is a potential target for a wide spectrum of antivenom drug development [18]. Inhibitors of these enzymes may be used as a first aid therapeutic against envenomation caused by several snake venoms. For example, Varespladib inhibited the PLA_2_ activity and, as such, reduced the lethal effects of *Protobothrops mucrosquamatus* snake venom in a mouse model [43].

### 2.2. Snake Venom Metalloproteinases

SVMPs are zinc-dependent proteinases with sizes ranging from 20–100 kDa and are classified into P-I, P-II, and P-III by their structural domains, with further divisions into subdomains [14,24,25]. The P-I SVMP has a metalloproteinase (M) domain, P-II SVMP has the M and disintegrin-like (D) domains, while the P-III SVMP has the M, D and cysteine-rich (C) domains [24]. These enzymes affect the coagulation cascades leading to the alteration of haemostatic balance, causing haemorrhage and induction of both local and systemic bleeding [24,44]. SVMPs hydrolyse vital constituents of the capillary vessel of the basement membrane components such as the type IV collagen, laminin and nidogen, leading to the weakening of the microvessels and other tissue components, thereby contributing to venom-induced local tissue damage [1,26]. The microvascular damage induced by SVMPs can be associated with the disruption of endothelial cell-cell adhesions and extracellular matrix proteins [44,45]. Peptidomimetic hydroxamate (Batimastat and Marimastat) metalloproteinase inhibitors were effective in abrogating local and systemic toxicity caused by *E. ocellatus* venoms [46].

### 2.3. Snake Venom Serine Proteases

SVSPs are monomeric glycoproteins with a molecular mass of 25–67 kDa and ~228–239 residues [14]. These toxins are primarily haemotoxic, which affects several physiological functions, including blood coagulation, platelet aggregation, fibrinolysis and blood pressure of envenomed victims [14,27]. SVSPs are found mainly in the venom of snakes from Viperidae and, to a lesser extent, in other families such as the Elapidae and Colubridae [47]. These enzymes possess a characteristic triad whereby a very reactive serine residue within the active site plays a key role in the formation of a transient acyl-enzyme complex stabilised by histidine and aspartate residues [48]. SVSPs can hydrolyse peptide bonds or esters and are specific to their substrates [49]. Those that display fibrinogenolytic activity are referred to as the snake venom thrombin-like enzymes [50]. They are functionally similar to thrombin as they can cleave the fibrinogen, causing coagulopathy and hypofibrinogenemia [47,50]. Inhibitors of snake venom serine proteases have been identified, including metabolites hesperetin (plant flavonoid) [51], synthetic peptides pepB (Pro-Phe-D-Arg-Gln-Ala-Ser) and pepC (Pro-Phe-Tyr-Gln-Ser-Ser) [52] that inhibited SVSP from *Bothrops jararaca*. These SVSP inhibitors can be used to improve envenomation treatment. For instance, the combination of serine protease inhibitors pepC and pepB with bothropic antivenom improved the latter’s overall performance through the reduction of haemorrhage and coagulopathy caused by *B. jararaca* venom [53].

### 2.4. Cysteine-Rich Secretory Proteins

CRISPs are secreted proteins mainly found in the mammalian epididymis, and granules appear to play roles in sperm epididymal maturation and immunity [54,55,56]. CRISPs have molecular weights of ~20–30 kDa and are strictly conserved cysteines [28]. Most of the conserved cysteine residues are clustered towards the C-terminal end of the proteins. The CRISPs have a hinge region that connects the cysteine-rich secretory proteins, antigen 5, and pathogenesis-related 1 proteins (CAP/PR-1) domain at the N-terminal with cysteine-rich (CRD)/ion channel regulatory (ICR) domain at the C-terminus [57]. Snake venom CRISPs toxicity includes blocking of potassium-stimulated smooth muscle contraction and cyclic nucleotide-gated ion channels [28]. These toxins have been identified in Viperidae and Elapidae from different continents, which shows a wide distribution of CRISP family proteins [54,58].

### 2.5. Three-Finger Toxins

Three-finger toxins (3FTxs) are non-enzymatic protein components of snake venom found mainly in the Elipdae with 60–74 amino acid residues containing three β strand loops linked to 4–5 disulphide bridges [29]. These toxins can be short-chain, long-chain or non-convectional [31]. A toxicovenomics study of snake venom from *Naja melanoleuca* revealed 3FTxs as the dominant protein component [59]. These toxin families have been reported to recognise several molecular targets such as nicotinic acid, muscarinic acetylcholine receptors and L-type channels and trigger various biological properties. Both type I and II α-neurotoxin target the nicotinic acetylcholine receptor (nAChR) at the end-plate of muscle fibre leading to flaccid paralysis, respiratory failure and death in the snake victim [60,61]. Cytotoxins of the 3FTxs family from the venom of *Naja nigricollis* induced a drastic dermonecrosis associated with early oedema of the dermis, blistering, loss of skin appendages and reduction in cellularity in a mouse model [30].

### 2.6. Other Toxins

The activities of other selected snake venom protein components, such as LAAO, CTL, DIS, KUN, and CYS, are shown in Table 1. LAAO is a flavin adenine dinucleotide-containing enzyme that catalyses the breakdown of L-amino acid into α-keto acid and releases ammonia and hydrogen peroxide as by-products [36]. LAAO has been linked to biological effects such as cytotoxic, platelet aggregation, haemorrhage, myonecrosis and oedema [36,62]. Additionally, the ability of snake venom LAAO to release hydrogen peroxide has made it a potential cytotoxic agent for anticancer therapeutic [36]. CTL are non-enzymatic proteins that bind to mono- and oligosaccharides in a Ca^2+^-dependent manner which plays a role in adhesion, endocytosis and pathogen neutralisation [32]. These proteins regulate haemostasis by altering blood coagulation and platelet aggregation [32,63]. DIS are cysteine-rich polypeptides with about 40–100 amino acids from snake venoms of the Viperidae family, which act as platelet aggregation inhibitors [34]. These non-enzymatic proteins released through proteolytic processing of multi-domain PII-SVMP precursors selectively block the β_1_ and β_3_ integrin receptors [34,64]. DIS inhibits integrin-mediated cell adhesion and cell migration. It is, therefore, a potential candidate for therapeutic tools as an anti-inflammatory and inhibitor of metastatic invasion [65].

KUN belongs to the functionally diverse bovine pancreatic trypsin inhibitor (BPTI) family characterised by a conserved fold of 50–60 amino acid residues cross-linked by three disulphide bridges [35,66]. They have been implicated in protease inhibition, anticoagulation, neurotoxicity, and blocking of neuronal voltage-dependent K^+^ and Ca^2+^ channel activities [35,38,67,68]. Snake venom CYS are cysteine protease inhibitor which affects blood coagulation and blood pressure [38]. CYS-like proteins that have been identified from snake venoms are closely related to type 2 cystatins with approximately 120 amino acid residues with two disulphide bonds [38,69,70]. It is envisaged that the snake venom cystatin could be protecting venom proteins from the proteolytic activities of the proteases from the snakebite victim or prey [37]. In this review, the abundance of cystatin was very low (minor class in Viperidae and amongst the “other” classes in the Elapidae). In vivo and in vitro studies showed that recombinant snake venom cystatin inhibited the growth, invasion and metastasis of tumour cells, highlighting its potential therapeutic application in cancer treatment [71].

## 3. Bottom-Up Snake Venom Proteomics

**Figure 1:**
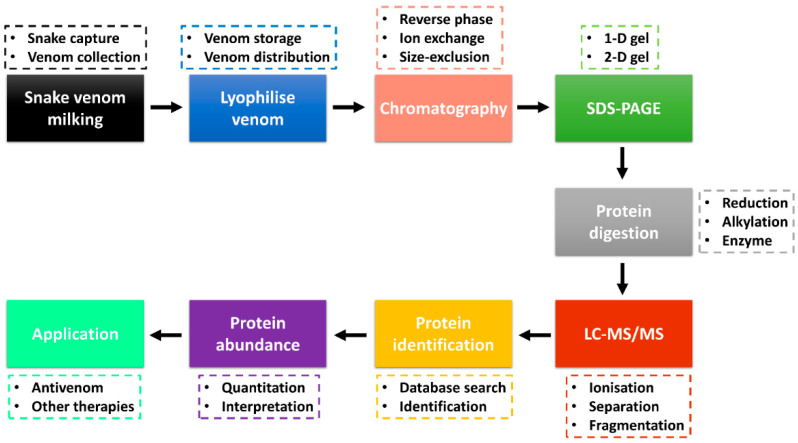
Snake venom bottom-up proteomics workflow through decomplexation strategy. Venom is collected through milking, lyophilised and the protein fractions are separated by chromatography and SDS-PAGE. The fractions are digested and analysed by LC-MS/MS, followed by protein identification. The venom proteome information is useful in the production of vaccines and other therapeutics. The decomplexation strategy was adapted from Tan et al. [72,80].

Advancements in the ‘omics’ technologies, especially proteomics, accompanied by databases and bioinformatics tools, have been excellent in the identification of protein toxins and analysis of their relative abundance [72,73]. Snake venom proteomics (bottom-up and/or top-down) has improved the high-throughput study of venom protein composition and other downstream venom studies, including envenomation, venom evolution, antivenom production and toxin-based drug discovery [10,72,74]. A combination of several techniques is being used in the study of snake venomics, with several analyses performed through the protein decomplexation strategy reported to increase protein identification [75]. Figure 1 shows the bottom-up snake venom proteomics with a decomplexation strategy and application of the obtained data information. Snake venom is collected through the milking process (e.g., manual milking) and stored at −20 °C or snap-frozen at −80 °C. In most cases, the venom is lyophilised to enhance its shelf life and easy distribution. Venom collection and lyophilisation is a critical step that requires care as it can affect downstream sample analysis and data interpretation. A moderate to a large amount (typical milligrams of proteins) is required to perform decomplexation venom proteomics.

The lyophilised snake venom can be separated into various fractions using chromatography techniques, including reverse-phase (RP), ion-exchange or size-exclusion high-performance liquid chromatography (HPLC). Most snake venom proteomics has adopted sample fractionation with the use of the C18 RP column coupled to the HPLC (RP-HPLC) [72,76,77]. The collected fractions are analysed through one-dimensional (1-D) or two-dimensional (2-D) sodium dodecyl sulphate-polyacrylamide gel electrophoresis (SDS-PAGE) [78]. In most decomplexation strategies, the reducing and/or non-reducing 1-D gel electrophoresis is used to analyse the fractions to identify fractions with protein bands that can be subjected to further LC-MS/MS analysis and protein identification. The pooled fractions and/or protein bands are reduced, alkylated and digested by the enzyme (e.g., trypsin, chymotrypsin) and analysed by LC-MS/MS. Two major ionisation techniques employed are matrix-assisted laser desorption ionisation (MALDI) and electrospray ionisation (ESI) [79]. The peptide fragments are searched against a reference database for protein identification. Using the fractionation chromatography peaks (e.g., RP-HPLC), the abundance of the protein families (estimated using the area under the curve) is calculated. The proteome information obtained can be used in the development of vaccines and antivenoms used in the treatment of snakebite envenoming and in other potential therapeutics for the treatment of other diseases such as cancer and coagulopathy [14,74].

## 4. Proteome Components of Venoms of African Viperidae and Elapidae

Snake venom comprises a mixture of proteins and peptides of different toxins. There are inter- and intraspecific variations of venom components due to the influence of genetic mutations, genetic drift, diet, season and natural selection that moulds the venom of each species for optimal adaptation [9,19]. Translational, transcriptional and post-translational modification control mechanisms play a key role in regulating the variability of venom toxins synthesis observed amongst related snake species [11]. Proteomic and immunological information on snake venoms provides clues on the mixture, clinical and evolutionary implication of venoms for the design of broad-spectrum antivenoms capable of neutralising the toxins [75].

Here, an online search was conducted using search engines such as Google (https://www.google.com/ accessed on 8 August 2022), Google Scholar (https://scholar.google.com/ accessed on 8 August 2022), PubMed (https://pubmed.ncbi.nlm.nih.gov accessed on 8 August 2022) and ScienceDirect (https://www.sciencedirect.com/ accessed on 8 August 2022) databases using the keywords “African snake venom proteome”, “African snake venomics”, “snake protein components”, and “African antivenoms”. Also, different African snake genera and species names were included in the search. The results were screened manually for the venom proteome data of Viperidae and Elapidae. Proteomic studies without quantified protein family abundance were excluded from the analyses. For comparison purposes, snake species with more than one proteomic data set were included in the analysis. For simplicity, the families that represent major protein abundance of total venom proteome are represented here.

**Figure 2:**
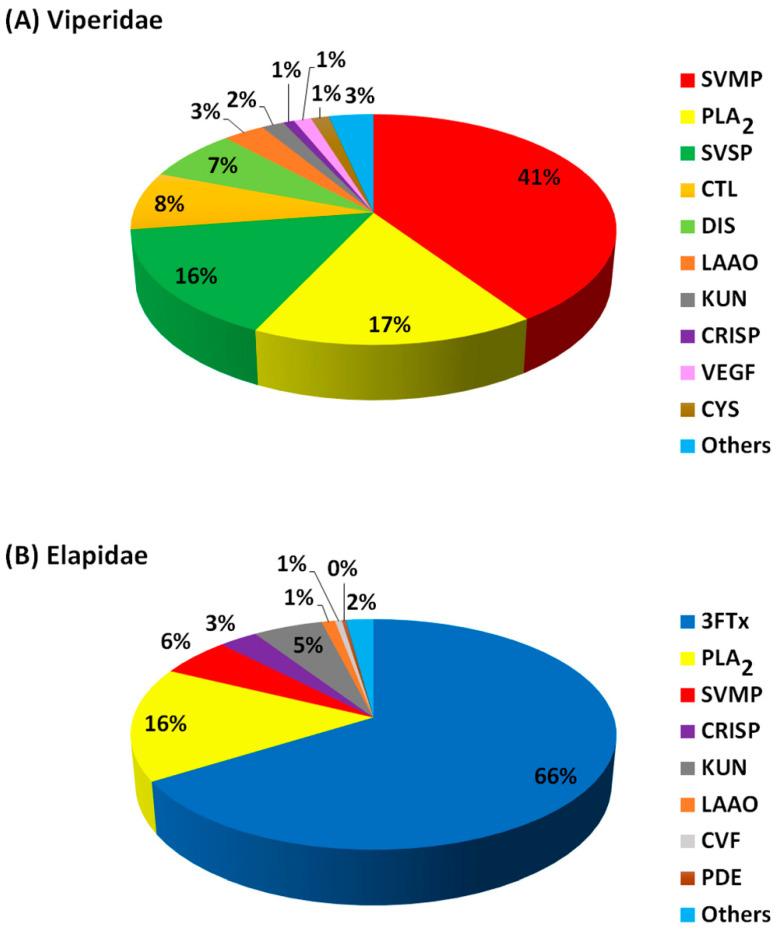
The proportion of the major families of the Viperidae (**A**) and Elapidae (**B**) snake venom protein components. Protein abundance values were averaged from the number of snake species studied. Abbreviations: PLA_2_ = phospholipase A_2_; SVMP = snake venom metalloproteinase; SVSP = snake venom serine protease; CTL = C-type lectin; DIS = disintegrin; LAAO = L-amino acid oxidase; KUN = Kunitz-type peptides; CRISP = cysteine-rich secretary protein; VEGF = vascular endothelial growth factor; CYS = cystatin; 3FTxs = three-finger toxins; CVF = cobra venom factor; PDE = endonucleases/phosphodiesterases.

The protein components (expressed as percentage abundance of the protein family in a venom) from snake venoms of Viperidae (Figure 2A; Table 2) and Elapidae (Figure 2B; Table 3) were analysed and classified into four groups (dominant, secondary, minor and others). Protein families in these three classes made up over 80% of the entire proteins in each venom. The percentage abundance of protein families in the “others” were mostly less than 2% in each snake venom, as summarised in Figure 2. The actual percentage abundant values for each of these “other” proteins are shown in Table S1 (Viperidae) and Table S2 (Elapidae). For Viperidae, 24 protein families were identified from 10 snake species (15 studies), while for Elapidae, 34 protein families were identified from 17 species (19 studies). The proteins in the former were grouped into ten major families, while the latter were grouped into eight major families. Overall, the most abundant protein families in Viperidae are SVMPs, followed by the PLA_2_ and SVSP, whereas 3FTxs and PLA_2_ were dominant in the Elapidae (Figure 2).

### 4.1. Dominant Viperidae Toxins

SVMPs, PLA_2_ and SVSPs are the most dominant proteins in the African Viperidae snake venom and thus are classified as dominant toxins (Figure 2A; Table 2). Amongst the dominant Viperidae toxins, SVMPs were the most abundant proteins, followed by the PLA_2_ and the SVSPs. These toxins make up an average of 73% of the Viperidae venom proteome. The proteome pattern is in agreement with several proteome studies of Viperidae from other continents and is believed to be the major player that causes snakebite fatalities observed in the victims [13,75]. The highest abundance of SVMPs, PLA_2_ and SVSPs was observed in *E. ocellatus* [81], *B. caudalis* [82] and *B. gabonica gabonica* [83]. Proteome profiles of two Nigerian Vipers, *B. arietans* and *E. ocellatus* showed that serine proteases (22.3%) and metalloproteinases (21.1%) dominated the venom of the former while metalloproteinases (34.8%), phospholipase A_2_s (21.2%) and serine proteases (15.5%) dominated the later [84]. Interestingly, 3FTxs that are dominant in the Viperidae were detected in the Elapidae as other toxins due to their lower abundance. Notably, 3FTxs families were not detected in the previous proteome studies of African Viperidae venom until recently [84].

**Table 2:** Percentage abundance of total protein for the major families in African Viperidae snake venom.

		Dominant			Secondary				Minor			Others	
Species	Country/Region	SVMP	PLA2	SVSP	CTL	DIS	LAAO	KUN	CRISP	VEGF	CYS	Others	Ref.
***B. arietans***	Ghana	38.5	4.3	19.5	13.2	17.8	–	4.1	–	–	1.7	0.9	[85]
***B. arietans***	Nigeria	21.1	10.1	22.3	10.7	3.4	8.7	1.1	2.1	8.1	–	8.6	[84]
***B. nasicornis***	West Africa	40.9	20.1	21.9	4.2	3.5	3.2	–	1.3	–	4.2	0.8	[82]
***B. caudalis***	West Africa	11.5	59.8	15.1	4.9	2.3	1.7	3.2	1.2	–	–	0.3	[82]
***B. g. gabonica***	East Africa	22.9	11.4	26.4	14.3	3.4	1.3	3	2	1	9.8	4.5	[83]
***B. g. rhinoceros***	West Africa	30.8	4.8	23.9	14.1	8.5	2.2	7.5	1.2	–	5.3	1.7	[82]
***E. ocellatus***	Nigeria	66.5	12.6	2	7	6.8	1.4	–	1.5	–	–	2.2	[81]
***E. ocellatus***	Nigeria	34.8	21.2	15.5	3.9	1.9	2.2	1.5	2.9	2.7		12.8	[84]
***C. cerastes***	Egypt	28.18	16.01	24.87	9.1	9.69	–	–	–	–	–	12.15	[86]
***C. cerastes***	Tunisia	37	20	9	24	8	12	–	–	–	–	–	[87]
***C. cerastes***	Tunisia	55.9	16.6	13.2	3.2	4.9	6.2	–	–	–	–	–	[88]
***C. cerastes***	Morocco	63.1	19.1	6.9	1.7	8.5	–	–	0.7	–	–	–	[88]
***C. vipera***	Tunisia	48	21	20	1	<1	9	–	–	–	–	–	[87]
***M. mauritanica***	Morocco	45.4	5.5	8.3	8.1	13.8	–	2.5	–	4.9	–	5.6	[88]
***M. lebetina***	Tunisia	63.1	5	5.5	3.2	15.1	–	3.1	–	3.3	–	1.7	[88]

Abbreviations: PLA_2_ = phospholipase A_2_; SVMP = snake venom metalloproteinase; SVSP = snake venom serine protease; CTL = C-type lectin; DIS = disintegrin; LAAO = L-amino acid oxidase; KUN = Kunitz-type peptides; CRISP = cysteine-rich secretary protein; VEGF = vascular endothelial growth factor; CYS = cystatin; B = *Bitis*; E = *Echis*; C = Cerastes; M = *Macroviperae* and – = not identified.

**Table 3:** Percentage abundance of total protein for the major families in African Elapidae snake venom.

		**Dominant**			**Secondary**		**Minor**			**Others**	
**Species**	Country/Region	3FTx	PLA_2_	SVMP	CRISP	KUN	LAAO	CVF	PDE	Others	Ref.
***D. polylepis***	Kenya	31	<0.1	3.2	–	61.1	–	–	0.1	4.5	[77]
***D. angusticeps***	Tanzania	69.2	–	6.7	2	16.3	–	–	–	6	[89]
***N. nigricollis***	Nigeria	73.3	21.9	2.4	0.2	–	–	–	–	2.2	[90]
***N. nigricollis***	Nigeria	41.25	36.5	7.91	3.4	–	4.02	1	–	3.24	[91]
***N. katiensis***	Burkina Faso	67.1	29	3.3	0.2	–	–	–	–	0.5	[90]
***N. pallida***	Kenya	67.7	30.1	1.6	–	–	–	–	–	0.7	[90]
***N. nubiae***	North Africa	70.9	26.4	2.6	–	–	–	–	–	0.1	[90]
***N. mossambica***	Tanzania	69.3	27.1	2.6	–	–	–	–	–	0.4	[90]
***N. melanoleuca***	Uganda	57.1	12.9	9.7	7.6	–	–	–	–	7.7	[59]
***N. annulifera***	Mozambique	78	–	11.18	0.61	–	5.01	1.08	–	2.53	[76]
***N. annulifera***	Mozambique	79.2	2.7	12.3	3.2	0.5	0.5	–	0.45	1.7	[92]
***N. ashei***	Kenya	69	27	2.1	0.7	–	–	0.12	–	1.014	[93]
***N. senegalensis***	West Africa	75.9	–	6.78	9.23	3.15	–	0.79	3.61	0.53	[94]
***N. haje***	Nigeria	52.14	24.02	7.2	4.85	–	3.63	3.93	–	3.85	[91]
***N. katiensis***	Nigeria	52.2	26	4.72	7	–	4.36	2.83	–	2.2	[91]
***H. haemachatus***	South Africa	63.3	22.8	7.1	4.1	1.5	–	–	–	0.6	[95]
***A. s. intermedius***	Southern Africa	82.7	6.1	2.9	4.9	1	–	–	0.4	1.88	[96]
***A. l. cowlesi***	Southern Africa	76.1	4.9	5.1	3.5	8.6	1	–	0.5	0.1	[96]
***A. l. lubricus***	Southern Africa	77.8	5.7	4	5.2	5.5	1	–	1.1	0.17	[96]

Abbreviations: 3FTxs = three-finger toxins; PLA_2_ = phospholipase A2; SVMP = snake venom metalloproteinase; CRISP = cysteine-rich secretary protein; KUN = Kunitz-type peptides; LAAO = L-amino acid oxidase; CVF = cobra venom factor; PDE = endonucleases/phosphodiesterases; D = *Dendroaspis*; N = *Naja*; H = *Haemachatus*; A = *Aspidelaps*; l = *lubricus*, s = *scutatus* and – = not identified.

### 4.2. Secondary Viperidae Toxins

The secondary toxins of the African Viperidae are CTL, DIS, LAAO and KUN, which averaged 20% of the venom proteome. Here, these toxin families were the second most dominant proteins. CTL is the second most abundant toxin (24%) in the Tunisian *Cerastes cerastes* snake venom but was, however, lower (1%) in the *C. vipera* species [87]. DIS is the third most abundant toxin in the proteome of Nigerian *E. ocellatus* and Ghanaian *B. arietans* [81,85], thus suggesting that DIS may play a key role in snakebite envenomation caused by this viper. Most LAAO enzymes were observed in the Tunisian *C. cerastes*, *C. vipera* and Nigerian *B. arietans* (Figure 2A; Table 2). KUN was identified in both *Bitis* and *Echis* genera of West Africa as well as in the North African *Macrovipera* snake venoms but was not identified in any of the *Cerastes* genera from North Africa. This variation can be attributed to geographical location differences.

### 4.3. Minor Viperidae Toxins

Minor toxin families are CRISP, VEGF and CYS, with a 4% average protein abundance in Viperidae (Figure 2A; Table 2). CRISP was detected (1–2.9%) in all the West and East African Viperidae snake venoms but was not identified in most of the North African *Macrovipera* and *Cerastes* genera, albeit (<1%) in the Moroccan *C. cerestas*. VEGF was fairly present in venoms of *Bitis*, *Echis* and *Macroviperan* genera but not in the *Cerestas*. CYS was identified in the *Bitis* genera only, which may be an indicator of intra- and inter-species variation in the Viperidae snake venoms.

### 4.4. Other Viperidae Toxins

These toxin families were present in low abundance and were grouped as ‘others’. They include snake venom natriuretic peptides (NPs), 5′-nucleotidase (5′N), three-finger toxins (3FTxs), nerve growth factor (NGF), bradykinin-potentiating peptides (BPP), peroxiredoxin, snake venom glutaminyl cyclases, secretory phospholipase A_2_ receptors, phospholipase B (PLB), phospholipase A_2_ inhibitor, globins, DC-fragment, SVMP inhibitors, peptides, and unknown (Table S1). All other Viperidae toxins had an average of 3% protein abundance. Most of these toxins were identified in the *Bitis* and *Echis* genera and most likely play various roles in snakebite envenomation.

### 4.5. Dominant Elapidae Toxins

These include 3FTxs, PLA2 and SVMPs, and make up an average of 87% of Elapidae venom (Figure 2B; Table 3). Overall, 3FTxs was the most abundant toxin, followed by the PLA_2_ and SVMPs. These three toxins were also reported as the most dominant proteins from Elapidae from other continents [19,75]. Surprisingly, PLA_2_ was not detected in the venom proteome of the Senegalese Cobra (*Naja senegalensis*) [94] and *Naja annulifera* (African cobra) [76], consistent with the unusual absence of PLA_2_ in the venoms of cobras in the *Uraeus* subgenus.

Similarly, PLA_2_ was not identified in the venom of *Dendroaspis angusticeps* [89] and with a very low abundance (<0.1) in *D. polylepis* [77], indicating a negligible role of this toxin in the toxicity of the mambas. Further analysis revealed the α-neurotoxin was largely present in the *D. polylepis* venom but was lacking in *D. angusticeps* [89]. It is interesting to note that the SVSP that was in the dominant toxins of the Vipridae were not identified in the Elapidae venoms. The proteomics analysis of the Ugandan forest cobra snake (*Naja melanoleuca*), amongst others, was dominated by 3FTx, which includes post-synaptically acting α-neurotoxin [59]. The dominant presence of 3FTx and PLA_2_ in the African spitting cobras may be the major contributor to the toxic effects of these venoms, such as local tissue necrosis [90]. Proteome analysis of the venoms of South African shielded-nosed or coral snakes (genus *Aspidelaps*) such as intermediate shield-nose snake (*Aspiderlaps scutatus intermedius*), Cowle’s shield snake (*Aspiderlaps lubricus cowlesi*) and Cape coral snake (*Aspiderlaps lubricus*) shows cross-genus dominance of 3FTxs and PLA_2_s with various isoforms of the former linked to interspecies variation. The proteome analysis of the venoms of three African spitting cobras (*H. haemachatus*, *N. mossambica* and *N. nigricollis*) and one non-spitting snouted cobra (*N. annulifera*) reported that while 3FTxs and PLA_2_s were the most abundant proteins in the spitting cobras, 3FTxs and SVMP were dominant in the non-spitting cobra, amongst others [92].

### 4.6. Secondary Elapidae Toxins

Secondary toxins comprise the CRISP and KUN family proteins which are made up of an average of 8% in Elapidae venom proteomes (Figure 2B; Table 3). In the Elapidae venom analysed, KUN had the highest abundance in the *D. polylepis* (61.1%) and the second most abundant in *D. angusticeps* (16.3%). This suggests that the KUN toxin contributes massively to the envenomation caused by the *Dendroaspis* genera. While *D. polylepis* venom was dominated by KUN, *D. angusticeps* was dominated by the 3FTxs. The toxin variations observed can be a result of dietary variations due to the divergent terrestrial ecology of black mamba (*D. polylepsis*) compared to the arboreal niche of green mambas (*D. viridis*, *D. angusticeps*, *D. jamesoni jamesoni* and *D. jamesoni kaimosae*) [97].

### 4.7. Minor Elapidae Toxins

The Elapidae minor toxins are LAAO, CVF and PDE, with an average of 2% for the venom protein families. These toxins were not identified in the *Haemachatus haemachatus* and Dendroaspis species, albeit 0.1% of PDE was observed in *D. polylepis* (Figure 2B; Table 3).

### 4.8. Other Elapidae Toxins

There are 27 toxin families in the ‘others’ group. These toxins include VEGF, 5′N, HYA, CTL, DIS, CYS, NGF, endonucleases, nawaprin, etc. All the toxin families in the ‘others’ group average 2% of the Elapidae venom proteome and are shown in Table S2. Interestingly, CTL, DIS, VEGF and CYS, which are a major part of Viperidae venom classes, were identified in the Elapidae as part of the ‘others’ group, suggesting that these toxins are more potent in the Viperidae compared to the Elapidae.

The major difference in the proteome of the Viperidae and Elapidae includes that 3FTxs and PLA_2_ dominated the proteome of the elapid’s venom, whereas SVMP, PLA_2_ and SVSPs dominated the viperid’s venom.

## 5. Antivenom for African Snakebite Envenomation

Antivenom remains the most effective and safe treatment for snakebite envenoming. Currently, there is a shortage of antivenom supply to Africa, which worsens the increase in snakebite morbidity and mortality within the continent. This shortage is caused by the high cost of production, cessation of production by the manufacturers, inefficient distribution and poor epidemiological information [98,99].

**Figure 3:**
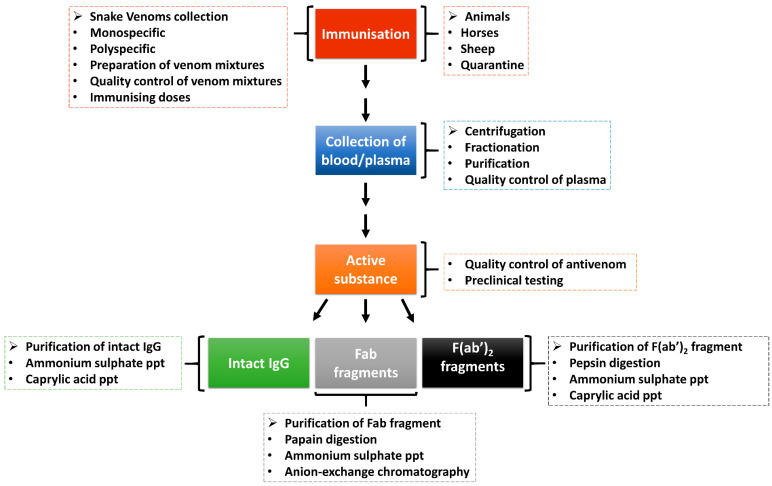
Production of antivenom. Animals are hyper-immunised with snake venoms, and the plasma is fractionated and purified to generate antivenoms in the form of intact immunoglobulin (IgG) or immunoglobulin fragments (Fab or F(ab)_2_). There are quality control steps in the antivenom production pipeline to ensure that it is of high quality and safe before usage (adapted from WHO, [100]).

Antivenom, which is the main treatment of snakebite envenomation, works by boosting the immune system of a victim after a snakebite. The body of the victim produces robust antibodies which target and neutralise the venom components and, as such, allow the body to eliminate the toxins. Figure 3 shows the process of antivenom production from animals immunised with snake venoms. To ensure the effectiveness and high-quality of antivenom, the WHO has adopted guidelines that include ensuring that the right venoms are used for immunisation and the animals used are healthy [100]. During the production of antivenom, several ethical considerations are observed, including the use of the venomous snake for the production of snake venoms, the use of large animals in the production of hyperimmune plasma, and the use of animals (usually mice in large quantities which also speaks to ethical concerns) in preclinical testing of antivenoms [100]. Nevertheless, there is an emphasis on the adoption of 3Rs concepts and principles as alternative assays to replace, refine and reduce the use of experimental animals in antivenom production and quality control processes [101].

Antivenoms are prepared through extraction and preparation of whole IgG, monovalent fragment antibody (Fab) or divalent fragment antibody F(ab’)2 from venom-immunised horses or other animals. The snake venom used as an immunogen in antivenom production is mixed with adjuvants to enhance the immune system response [102]. While creating an adequate venom immunisation mixture, it is important to take into account different geographical origins, age, sex, diet, season and intra-specific venom variations of the medically important snakes within the country, region or continent targeted for antivenom production [100]. The antivenom is a monospecific antivenom when it is generated from a single venomous snake species or polyspecific antivenoms when from several species. The polyspecific antivenom has more advantages as it is capable of neutralising the effect of several snake venoms from different species and geographical locations [103].

The animals (e.g., horses or sheep) are quarantined and vaccinated against specific diseases and treated against diseases [102]. The animals are then introduced to an immunisation programme with venom doses. Plasma is separated from the whole blood through centrifugation or sedimentation and is quality-checked before fractionation. The red blood cells can be reinfused into the animal after separation. The resultant active substance is produced as intact immunoglobulins (IgG) or enzymatically digested into immunoglobulin Fab or F(ab’)_2_ fragments [102,104,105]. These antivenoms are purified through ammonium sulphate or caprylic acid precipitation to remove non-immunoglobulin proteins [104,106]. Further purification can be achieved using chromatographic techniques to enhance purity [104]. The antivenoms are then subjected to quality control and preclinical and clinical testing for safety and efficacy before usage/supply [102].

Table 4 outlines selected antivenoms commonly used in the treatment of snakebite envenoming in Africa with emphasis on their cross-reactivity and/or cross-neutralisation capacity on snake venoms. Since 1971, South African Vaccine Producers (SAVP) (formerly South African Institute of Medical Research (SAIMR)) produced polyvalent snake antivenom prepared from the serum of hyper-immunised horses with the venom of ten southern African snake species and has been the main antivenom used in Southern Africa [107]. An in vitro study showed that SAIMR polyvalent antivenom (0.11–0.53 µg/µL of total protein) was effective in neutralising the toxic effects of *B. arietans* venom in the L6 rat skeletal muscle cells [108]. However, the venom of South African shielded-nosed or coral snakes (genus *Aspidelaps*) was not included in the venoms used to immunise horses for the manufacturing of regional SAIMR polyvalent antivenom due to the snake’s uncommon bites. Nevertheless, the antivenom was effective in neutralising the venoms from the *A. s. intermidius*, *A. l. cowlesi* and *A. l. lubricus*, comparable to those observed in the cape cobra (*N. nivea*), suggesting a wider than anticipated clinical utility of the antivenom [96].

A whole IgG polyspecific EchiTAb-Plus-ICP antivenom designed for the treatment of snakebite envenomings in sub-Saharan Africa was effective in neutralising Viperid venoms for both *Echis* sp. (*E. ocellatus*, *E. leucogaster*, *Echis pyramidum leakeyi*) and *Bitis* spp. (*B. arietans*, *B. gabonica*, *B. rhinoceros and B. nasicornis*) from Nigeria and other sub-Saharan African locations [109]. The EchiTAb-Plus-ICP antivenom was developed for the treatment of snake bite envenoming by immunising horses with venoms of sub-Saharan Africa medically important snake species (*E. ocellatus*, *B. arietans and N. nigricollis*) [110]. This antivenom immunodepleted the majority of venom proteins, including SVMPs, SVSPs, CTL proteins, some PLA_2_ and LAAO in snake venoms of *Echis* and *Bitis* genera [111]. In another study, the Pan-Africa EchiTAb-Plus-ICP antivenom neutralised the lethal, dermonecrotic and PLA_2_ activities of *N. nigricollis* and *N. mossambica*, lethal and PLA_2_ activities of *N. palliada* and finally, the PLA_2_ activities of *N. katiensis* and *N. nubiae* [90]. The expansion of neutralising effect of West African-used EchiTAb-Plus-ICP was achieved through the addition of some of the most medically important elapids (*B. arietans*, *N. mossambica*, *N. annulifera*, *H. haemachatus*) from the southern region (Swaziland) to obtain expanded-scope antivenom called EchiTAb-P + ICP antivenom [112].

The hetero-specific VINS African Polyvalent Antivenom (VAPAV) was immunoreactive and cross-neutralised the venom of *N. senegalensis* (predominated with cardiotoxin/cytotoxin) and α-neurotoxin, with no PLA_2_) [94]. It showed strong neutralising capacity against four African Viperid (*E. leucogaster*, *E. ocellatus*, *B. arietans*, *B. gabonica*) and six African Elapids (*D. jamesoni*, *D. polylepis*, *D. viridis*, *N. haje*, *N. melanoleuca*, *N. nigricollis*) snake species [113]. Both the premium serum pan Africa polyvalent antivenom (PANAF) and VAPAV were immunoreactive and cross-neutralised the lethal effect of *N. annulifera* venom, with PANAF being marginally more potent [76]. *D. polylepis* and *D. angusticeps* venoms were neutralised by three polyvalent antivenoms: (a) SAIMR antivenom from South African vaccine producers, (b) Snake venom antivenom (Central Africa) and (c) Snake venom antivenom (Africa) from VINS Bioproducts Ltd. [77,89]. In this regard, SAIMR, VINS African and VINS Central Africa neutralised the lethality of *D. polylepis* venom, albeit in different potencies [77,97]. SAIMR and VINS African antivenoms neutralised the lethal effect of *D. angusticeps* venom, while the VINS central African antivenom failed to neutralise the lethality of the venom with the lowest antivenom ratio tested (1.0 mg venom/mL) [89]. Notably, *D. angusticeps* was not included in the immunisation mixture used in the production of the VINS central African antivenom.

The monospecific anti-*Hemachatus*-ICP antivenom produced by Costa Rican Instituto Clodomiro Picado was able to neutralise the toxicity and lethality of three spitting cobras (*N. mossambica*, *N. nigricollis* and *H. haemachatus*). Nevertheless, it was not effective against the toxicity and lethality of the non-spitting cobra, *N. annulifera*. This suggests a closer relationship between *Haemachatus* and *Naja* spitting cobra than the *Naja* non-spitting cobra [92]. Monospecific anti-Moroccan *C. cerastes* (CcMo_AV) and the Gamma-VIP divalent antivenom had similar immunocapturing capability toward snake venom proteins of *C. cerastes* from Morocco, Tunisia and Egypt [88]. In addition, the anti-Moroccan *M. mauritanica* (MmMo_AV) and commercially available Gamma-VIP divalent antivenom show great immunoreactivity towards Moroccan *M. mauritanica* and Tunisian *M. labetina* venoms [114].

*E. ocellatus* venoms from Mali, Cameroon and Nigeria were neutralised by antivenoms distributed in sub-Saharan Africa, such as EchiTAb-Plus-ICP, EchiTAb-G, FAV Afrique and Inoserp Panafricain antivenoms [115]. Potet et al. [116] reported EchiTAb-Plus, EchiTAb-G and SAIMR monovalent as vaccines that have been well-clinically tested and shown to be effective against envenomation caused by *E. ocellatus*. A preclinical efficacy study comparing four tests (PANAF, VINS African, Inoserp Panafricain, FAV Afrique) and two gold standard antivenoms (SAIMR polyvalent and SAIMR Echis carinatus) against medically important snake venoms in East Africa revealed PANAF as the most effective and affordable of the tested antivenoms [117]. The overall variation and low venom-neutralising efficacies of the antivenoms suggested the possibility that these antivenoms were produced with venoms from different geographical locations since none of the producers stated the geographical origin of the snakes.

There is a need to intensify clinical testing for all antivenoms in the African market space and also advance research to develop African-specific antivenoms with the most medically important snake venoms from all regions, which will alleviate snakebite envenomation within the continent.

**Table 4:** Selected antivenoms used in Africa.

Antivenom	Active Substance	Antivenom Producing Company	Venom Used for Immunisation		Venom Cross-Neutralisation		Ref.
			Viperidae	Elapidae	Viperidae	Elapidae	
EchiTAb-Plus-ICP	IgG	Instituto Clodomiro Picado, Costa Rica	*E. ocellatus*, *B. arietans*	*N. nigricollis*	*E. ocellatus*, *E. leucogaster*, *E. pyramidium leakeyi*, *B. arietans*, *B. gabonica*, *B. rhinoceros*, *B. nasicornis*	*D. poylepis*, *N. nigricollis*, *N. mosaambica*, *N. annulifera*, *N. nubiae*, *N. katiensis*, *N. pallida*, *H. haemachatus*	[90,92,109,110,111,112,118]
EchiTAb-Plus + ICP (expanded)	IgG	Instituto Clodomiro Picado, Costa Rica	*E. ocellatus*, *B. arietans*	*D. poylepis*, *N. nigricollis*, *N. mosaambica*, *N. annulifera*, *H. haemachatus*	*E. ocellatus*, *B. arietans*	*D. polylepis*, *N. mossambica*, *N. annulifera*, *N. nigricollis*, *H. haemachatus*	[112]
FAV Afrique	F (ab’)_2_	Sanofi-Pasteur, France	*E. ocellatus*, *E. leucogaster*, *B. arietans*, *B. gabonica*	*D. polylepis*, *D. jamesoni*, *D. viridis*, *N. nigricollis*, *N. haje*	*B. arietans*, *E. ocellatus*	*D. polylepis*, *N. mossambica*, *N. annulifera*, *H. haemachatus*	[112,115]
ASNA antivenom C (ASNA-C)	F (ab’)_2_	Bherat Serum and Vaccines, India	*E. carinatus*, *B. arietans*, *B. gabonica*, *B. nasiconrnis*	*D. polylepis*, *D. jamesoni*, *D. angusticeps*, *N. gigricollis*, *N. annulifera*, *N. nivea*	*B. arietans*	*D. polylepis*, *N. mossambica*, *N. annulifera*, *H. haemachatus*	[112]
South African Institute for Medical research (SAIMR) Polyvalent	F (ab’)_2_	SAVP, South Africa	*B. arietans*, *B. gabonica*	*D. polylepis*, *D. jamesoni*, *D. angusticeps*, *N. melanoleuca*, *N. nivea*, *N. annulifera*, *N. mossambica*, *H. haemachatus*	*B. arietans*	*D. angusticeps*, *D. polylepis*, *N. mossambica*, *N. annulifera*, *H. haemachatus*, *A. s. intermedius*, *A. l. cowlesi*, *A. l. lubricus*	[59,89,96,97,108,112]
Snake venom antiserum (Central African) antivenom	F (ab’)_2_	VINS Bioproducts, India	*B. g. rhinoceros*, *Vipera russelli*, *E. carinatus*	*D. polylepis*		*D. angusticeps*, *D. polylepis*	[77,89,97]
Premium serum Pan African polyvalent antivenom (PANAF)	F (ab’)_2_	Premium Serum and Vaccines, India	*B. arietans*, *B. gabonica*, *B. nasicornis*, *B. rhinoceros*, *E. leucogaster*, *E. ocellatus*, *E. carinatus*	*D. jamesoni*, *D. polylepis D. viridis*, *D. angusticeps*, *N. haje*, *N. melanoleuca*, *N. nigrocollis*		*N. annulifera*	[76]
VINS African polyvalent antivenom (VAPAV)	F (ab’)_2_	VINS Bioproducts, India	*B. arietans*, *B. gabonica*, *E. leucogaster*, *E. ocellatus*	*D. jamesoni*, *D. polylepis D. viridis*, *N. haje*, *N. melanoleuca*, *N. nigrocollis*	*B. arietans*, *B. gabonica*, *E. leucogaster*, *E. ocellatus*	*D. polylepis*, *D. angusticeps*, *D. jamesoni*, *D. viridis*, *N. annulifera*, *N. senegalensis*, *N. haje*, *N. mossambica*, *N. nigricollis*, *N. melanoleuca*, *H. haemachatus*	[76,77,89,94,97,112,113]
EchiTAb G	IgG	MicroPharm, UK	*E. ocellatus*		*E. ocellatus*		[115]
Inoserp-Panafricain (Inoserp-P)	F (ab’)_2_	INOSAN Biopharma, Spain	*E. ocellatus*, *E. leucogaster*, *E. pyramidium*, *B. arietans*, *B. gabonica*	*D. polylepis*, *D. jamesoni*, *N. nigricollis*, *N. melanoleuca*, *N. haje*, *N. pallida*	*E. ocellatus*		[115]
anti-Hemachatus-ICP		Instituto Clodomiro Picado, Costa Rica		*H. haemachatus*		*H. haemachatus*, *N. mossambica*, *N. nigricollis*, *N. annulifera*	[92]
Anti-Moroccan *C. cerastes* antivenom (CcMo_AV)	F (ab’)_2_	Instituto Butantan, Brazil	*C. cerastes*		*C. cerastes*		[88]
Gamma-VIP	F (ab’)_2_	Institut Pasteur de Tunis, Tunisia	*C. cerastes*, *M. lebetina*		*C. cerastes*, *M. mauritanica*, *M. lebetina*		[88,114]
Anti-Moroccan *M. mauritanica* antivenom (MmMo_AV)	F (ab’)_2_	Instituto Butantan, Brazil	*M. mauritanica*		*M. mauritanica*, *M. lebetina*		[114]

## 6. Alternative Antivenom Therapy

The immunoglobulin-based antivenom produced from livestock has lots of drawbacks, including allergic reactions and hypersensitive reactions in patients, batch variability, lack of specificity and complex manufacturing process [119]. Alternative approaches have been explored to improve the current limitations of antivenom therapy. These include the development of toxin-specific antibodies via immunisation with DNA, synthetic epitope string immunogen and recombinant toxins [120,121]. Recombinant IgGs, Fabs, and single-chain variable fragments (scFVs) antibodies have been expressed in mammalian and *E. coli* host systems within antivenom research [122]. Optimisation of these expression systems and engineered expression vectors has led to improved yields and to achieve a higher degree of correct folding [122].

Sequences encoding the epitopes of venom toxin (SVMP) cDNA library obtained from an expressed sequence tag database and engineered into a single synthetic multiepitope DNA immunogen (a string of SVMP epitopes) neutralised haemorrhage induced by *E. ocellatus* and *C. cerastes* venoms [121]. Lethal toxin neutralisation factor (LTNF) peptide isolated from opossum (*Didelphis virginiana*) serum inhibited the lethality of snake venoms [123]. The *E. coli* expression of this short peptide yielded a recombinant LTNF that neutralised rattlesnake (*Crotalus atrox*) venom [124]. A combination of SVMP inhibitor marimastat and PLA_2_ inhibitor Varespladib was effective in counteracting the lethal effects of vipers from South Asia, sub-Saharan Africa and Central America [125]. Next-generation antivenoms may be achieved through hybrid products containing mixtures of antibodies (e.g., anti-3FTx, anti-PLA_2_, anti-SVSP, anti-SVMP), antibody fragments and small molecule inhibitors (e.g., marimastat and varespladib) [9,125]. Two peptidomimetic hydroxamate metalloproteinase inhibitors, such as Maristastat and Batimastat, reduced the lethality of *E. ocellatus* venoms from Cameroon and Ghana in the mouse model [46]. PLA_2,_ 3FTx, SVSP and SVMP are the major snake venom toxin targeted for next-generation therapeutics due to their dominant abundance and major pathological implication in snakebite victims [25]. This mixture, not based on only one antitoxin format, is proposed to enhance broad toxin neutralisation across distinct snake venoms. More studies are required to evaluate the efficacy, dosages, and route of administration of these alternative therapies. Nevertheless, alternative therapies are promising future drug leads for broad-spectrum therapeutics for the treatment of snakebite envenomation [125].

## 7. Concluding Remarks

Snakebite envenomation is an NTD that massively affects poorer communities and causes morbidity and mortality. There are species- and genus-specific variations in the toxins of snake venom, which leads to distinct toxicity and pathological effects thereof. Proteomic profiling of the medically important African snake venoms will contribute to the production of safer and more effective antivenom to treat snakebite envenomation in Africa. There is a low supply of antivenom in Africa, which needs to be upscaled to alleviate the effect of snakebite envenomation. The data obtained from proteome analyses of the African Viperidae and Elapidae snake venoms showed that SVMP, PLA_2_ and SVSP were the most abundant proteins in the former, while 3FTxs and PLA_2_ were the dominant protein families in the latter. Protein components were classified as dominant, secondary, minor and other toxins based on the toxin abundance in the venoms. We also reviewed the activities of selected antivenom used in the treatment of snakebite envenomation in Africa. In conclusion, the African Viperidae and Elapidae snake venoms have diverse protein components that can be used to develop more robust antivenom in the treatment of snakebite envenomation and as prospective therapeutic treatment of other diseases.

## Figures and Tables

**Table 1:** Summary of the biological activities of the major snake venom protein components found in Viperidae and Elapidae.

Protein Family	MW (kDa)	Description	Mode of Action	Elapidae	Viperidae	Ref.
PLA_2_s	13–15	Lipolytic enzymes hydrolyse ester bonds at the *sn*-2 position of the glycerophospholipids to release lysophospholipids and fatty acids.	Cause neurotoxicity, cytotoxicity, myotoxicity, cardiotoxicity, oedema and anticoagulant effects	V	V	[18,23]
SVMPs	20–100	Zinc-dependent proteinases	Hydrolyse vital constituents of the capillary vessel of the basement membrane leading to the weakening of the microvessels and local tissue damage	V	V	[14,24,25,26]
SVSPs	25–67	Monomeric or multimeric glycoproteins	Haemotoxic enzymes that affect coagulation factors, fibrinolysis, plasminogen or platelets	●	V	[14,27]
CRISP	20–30	Secreted proteins containing conserved cysteine mainly at the C-terminal	Has both smooth muscle contraction and cyclic nucleotide-gated ion channel-blocking activities	V	V	[28]
3FTxs	6–9	Non-enzymatic secreted protein superfamily of small toxins	Inhibit neuromuscular transmission by targeting nicotinic acid and muscarinic acetylcholine receptors, ion channels and membranes	V	●	[29,30,31]
CTL	13–15	Non-enzymatic protein with carbohydrate recognition domain	Prevents clotting and affects platelet activity	●	V	[19,32]
DIS	5–10	Inhibitors of integrin	Alters activity of platelet and promotes haemorrhage	●	V	[33,34]
KUN	6–7	Members of serine protease inhibitors	Causes neurotoxicity and disrupts haemostasis	V	V	[35]
LAAO	50–70	Converts L-amino acid into alpha-keto acid, release hydrogen peroxide and ammonia	Causes cell damage	V	V	[36]
CYS	12–13	Protease inhibitor family alters prey homeostasis	Affects blood coagulation and blood pressure	V	V	[37,38]

Abbreviations: PLA_2_ = phospholipase A_2_; SVMPs = snake venom metalloproteinases; SVSPs = snake venom serine proteases; CRISP = cysteine-rich secretary protein; 3FTxs = three-finger toxins; CTL = C-type lectin; DIS = disintegrin; KUN = Kunitz-type peptides; LAAO = L-amino acid oxidase; CYS = cystatin (V = present and ● = absent).

## Data Availability

The data presented in this study are available in this article or Supplementary Material.

## Supplementary Materials

The following supporting information can be downloaded at: https://www.mdpi.com/article/10.3390/toxins14110723/s1, Table S1: Percentage abundance of other venomic protein components of African Viperidae snakes; Table S2: Percentage abundance of other venomic protein components of African Elapidae snakes.

## References

[1] Gutiérrez J.M., Calvete J.J., Habib A.G., Harrison R.A., Williams D.J., Warrell D.A. (2017). Snakebite envenoming. Nat. Rev. Dis. Prim..

[2] Harrison R.A., Hargreaves A., Wagstaff S.C., Faragher B., Lalloo D.G. (2009). Snake envenoming: A disease of poverty. PLoS Negl. Trop. Dis..

[3] Chippaux J.P. (1998). Snake-bites: Appraisal of the global situation. Bull. World Health Organ..

[4] Kasturiratne A., Wickremasinghe A.R., De Silva N., Gunawardena N.K., Pathmeswaran A., Premaratna R., Savioli L., Lalloo D.G., De Silva H.J. (2008). The global burden of snakebite: A literature analysis and modelling based on regional estimates of envenoming and deaths. PLoS Med..

[5] WHO (2019). Snakebite Envenoming: Strategy for Prevention and Control.

[6] Chippaux J.P. (2011). Estimate of the burden of snakebites in sub-Saharan Africa: A meta-analytic approach. Toxicon.

[7] Halilu S., Iliyasu G., Hamza M., Chippaux J.P., Kuznik A., Habib A.G. (2019). Snakebite burden in sub-Saharan Africa: Estimates from 41 countries. Toxicon.

[8] Chippaux J.P. (2017). Snakebite envenomation turns again into a neglected tropical disease!. J. Venom. Anim. Toxins Incl. Trop. Dis..

[9] Casewell N.R., Jackson T.N.W., Laustsen A.H., Sunagar K. (2020). Causes and consequences of snake venom variation. Trends Pharmacol. Sci..

[10] Casewell N.R., Wüster W., Vonk F.J., Harrison R.A., Fry B.G. (2013). Complex cocktails: The evolutionary novelty of venoms. Trends Ecol. Evol..

[11] Casewell N.R., Wagstaff S.C., Wus̈ter W., Cook D.A.N., Bolton F.M.S., King S.I., Pla D., Sanz L., Calvete J.J., Harrison R.A. (2014). Medically important differences in snake venom composition are dictated by distinct postgenomic mechanisms. Proc. Natl. Acad. Sci. USA.

[12] Currier R.B., Harrison R.A., Rowley P.D., Laing G.D., Wagstaff S.C. (2010). Intra-specific variation in venom of the African Puff Adder (*Bitis arietans*): Differential expression and activity of snake venom metalloproteinases (SVMPs). Toxicon.

[13] Damm M., Hempel B.F., Süssmuth R.D. (2021). Old world vipers-a review about snake venom proteomics of viperinae and their variations. Toxins.

[14] Oliveira A.L., Viegas M.F., da Silva S.L., Soares A.M., Ramos M.J., Fernandes P.A. (2022). The chemistry of snake venom and its medicinal potential. Nat. Rev. Chem..

[15] Lu Q., Clemetson J.M., Clemetson K.J. (2005). Snake venoms and hemostasis. J. Thromb. Haemost..

[16] Williams D.J., Faiz M.A., Abela-Ridder B., Ainsworth S., Bulfone T.C., Nickerson A.D., Habib A.G., Junghanss T., Fan H.W., Turner M. (2019). Strategy for a globally coordinated response to a priority neglected tropical disease: Snakebite envenoming. PLoS Negl. Trop. Dis..

[17] Benjamin J.M., Abo B.N., Brandehoff N. (2020). Review article: Snake envenomation in Africa. Curr. Trop. Med. Rep..

[18] Xiao H., Pan H., Liao K., Yang M., Huang C. (2017). Snake Venom PLA2, a promising target for broad-spectrum antivenom drug development. Biomed Res. Int..

[19] Tasoulis T., Isbister G.K. (2017). A review and database of snake venom proteomes. Toxins.

[20] Slagboom J., Mladić M., Xie C., Kazandjian T.D., Vonk F., Somsen G.W., Casewell N.R., Kool J. (2020). High throughput screening and identification of coagulopathic snake venom proteins and peptides using nanofractionation and proteomics approaches. PLoS Negl. Trop. Dis..

[21] Koh C.Y., Kini R.M. (2012). From snake venom toxins to therapeutics—Cardiovascular examples. Toxicon.

[22] McCleary R.J.R., Kini R.M. (2013). Non-enzymatic proteins from snake venoms: A gold mine of pharmacological tools and drug leads. Toxicon.

[23] Six D.A., Dennis E.A. (2000). The expanding superfamily of phospholipase A2 enzymes: Classification and characterization. Biochim. Biophys. Acta.

[24] Kang T.S., Georgieva D., Genov N., Murakami M.T., Sinha M., Kumar R.P., Kaur P., Kumar S., Dey S., Sharma S. (2011). Enzymatic toxins from snake venom: Structural characterization and mechanism of catalysis. FEBS J..

[25] Ferraz C.R., Arrahman A., Xie C., Casewell N.R., Lewis R.J., Kool J., Cardoso F.C. (2019). Multifunctional toxins in snake venoms and therapeutic implications: From pain to hemorrhage and necrosis. Front. Ecol. Evol..

[26] Escalante T., Shannon J., Moura-da-Silva A.M., María Gutiérrez J., Fox J.W. (2006). Novel insights into capillary vessel basement membrane damage by snake venom hemorrhagic metalloproteinases: A biochemical and immunohistochemical study. Arch. Biochem. Biophys..

[27] Kini R.M. (2006). Serine proteases affecting blood coagulation and fibrinolysis from snake venoms. Pathophysiol. Haemost. Thromb..

[28] Yamazaki Y., Morita T. (2004). Structure and function of snake venom cysteine-rich secretory proteins. Toxicon.

[29] Frangieh J., Rima M., Fajloun Z., Henrion D., Sabatier J.M., Legros C., Mattei C. (2021). Snake venom components: Tools and cures to target cardiovascular diseases. Molecules.

[30] Rivel M., Solano D., Herrera M., Vargas M., Villalta M., Segura Á., Arias A.S., León G., Gutiérrez J.M. (2016). Pathogenesis of dermonecrosis induced by venom of the spitting cobra, *Naja nigricollis*: An experimental study in mice. Toxicon.

[31] Kini R.M., Doley R. (2010). Structure, function and evolution of three-finger toxins: Mini proteins with multiple targets. Toxicon.

[32] Ogawa T., Chijiwa T., Oda-Ueda N., Ohno M. (2005). Molecular diversity and accelerated evolution of C-type lectin-like proteins from snake venom. Toxicon.

[33] Kamiguti A.S., Zuzel M., Theakston R.D.G. (1998). Snake venom metalloproteinases and disintegrins: Interactions with cells. Braz. J. Med. Biol. Res..

[34] Calvete J.J. (2013). The continuing saga of snake venom disintegrins. Toxicon.

[35] Schweitz H., Heurteaux C., Bois P., Moinier D., Romey G., Lazdunski M. (1994). Calcicludine, a venom peptide of the Kunitz-type protease inhibitor family, is a potent blocker of high-threshold Ca^2+^ channels with a high affinity for L-type channels in cerebellar granule neurons. Proc. Natl. Acad. Sci. USA.

[36] Tan K.K., Bay B.H., Gopalakrishnakone P. (2018). L-amino acid oxidase from snake venom and its anticancer potential. Toxicon.

[37] Mashiko H., Takahashi H. (2002). Cysteine proteinase inhibitors in elapid and hydrophiid snake venoms. Toxicon.

[38] Inagaki H. (2017). Snake venom proteases inhibitors: Enhanced identification, expanding biological function, and promising future. Snake Venoms, Toxinology.

[39] Hiu J.J., Yap M.K.K. (2020). Cytotoxicity of snake venom enzymatic toxins: Phospholipase A2 and L-amino acid oxidase. Biochem. Soc. Trans..

[40] Cendron L., Mičetić I., Polverino De Laureto P., Paoli M. (2012). Structural analysis of trimeric phospholipase A2 neurotoxin from the Australian taipan snake venom. FEBS J..

[41] Wang C.R., Bubner E.R., Jovcevski B., Mittal P., Pukala T.L. (2020). Interrogating the higher order structures of snake venom proteins using an integrated mass spectrometric approach. J. Proteom..

[42] Mora-Obando D., Fernández J., Montecucco C., Gutiérrez J.M., Lomonte B. (2014). Synergism between basic Asp49 and Lys49 phospholipase A2 myotoxins of viperid snake venom in vitro and in vivo. PLoS ONE.

[43] Liu C.C., Wu C.J., Hsiao Y.C., Yang Y.H., Liu K.L., Huang G.J., Hsieh C.H., Chen C.K., Liaw G.W. (2021). Snake venom proteome of Protobothrops mucrosquamatus in Taiwan: Delaying venom-induced lethality in a rodent model by inhibition of phospholipase A2 activity with varespladib. J. Proteom..

[44] Seo T., Sakon T., Nakazawa S., Nishioka A., Watanabe K., Matsumoto K., Akasaka M., Shioi N., Sawada H., Araki S. (2017). Haemorrhagic snake venom metalloproteases and human ADAMs cleave LRP5/6, which disrupts cell–cell adhesions in vitro and induces haemorrhage in vivo. FEBS J..

[45] Gutiérrez J.M., Escalante T., Rucavado A., Herrera C., Fox J.W. (2016). A comprehensive view of the structural and functional alterations of extracellular matrix by snake venom metalloproteinases (SVMPs): Novel perspectives on the pathophysiology of envenoming. Toxins.

[46] Arias A.S., Rucavado A., Gutiérrez J.M. (2017). Peptidomimetic hydroxamate metalloproteinase inhibitors abrogate local and systemic toxicity induced by *Echis ocellatus* (saw-scaled) snake venom. Toxicon.

[47] Serrano S.M.T., Maroun R.C. (2005). Snake venom serine proteinases: Sequence homology vs. substrate specificity, a paradox to be solved. Toxicon.

[48] Barrett A.J., Rawlings N.D. (1995). Families and clans of serine peptidases. Arch. Biochem. Biophys..

[49] Serrano S.M.T. (2013). The long road of research on snake venom serine proteinases. Toxicon.

[50] Ullah A., Masood R., Ali I., Ullah K., Ali H., Akbar H., Betzel C. (2018). Thrombin-like enzymes from snake venom: Structural characterization and mechanism of action. Int. J. Biol. Macromol..

[51] Dos Santos R.V., Grillo G., Fonseca H., Stanisic D., Tasic L. (2021). Hesperetin as an inhibitor of the snake venom serine protease from *Bothrops jararaca*. Toxicon.

[52] Da Silva G.M., de Souza D.H.B., Waitman K.B., Ebram M.C., Fessel M.R., Zainescu I.C., Portaro F.C., Heras M., de Andrade S.A. (2021). Design, synthesis, and evaluation of Bothrops venom serine protease peptidic inhibitors. J. Venom. Anim. Toxins Incl. Trop. Dis..

[53] Silva G.M., Berto D.H., Lima C.A., Waitman K.B., Lima C.F.G., Prezoto B.C., Vieira M.L., Rocha M.M.T., Gonçalves L.R.C., Andrade S.A. (2021). Synergistic effect of serine protease inhibitors and a bothropic antivenom in reducing local hemorrhage and coagulopathy caused by *Bothrops jararaca* venom. Toxicon.

[54] Yamazaki Y., Hyodo F., Morita T. (2003). Wide distribution of cysteine-rich secretory proteins in snake venoms: Isolation and cloning of novel snake venom cysteine-rich secretory proteins. Arch. Biochem. Biophys..

[55] Kjeldsen L., Cowland J.B., Johnsen A.H., Borregaard N. (1996). SGP28, a novel matrix glycoprotein in specific granules of human neutrophils with similarity to a human testis-specific gene product and to a rodent sperm-coating glycoprotein. FEBS Lett..

[56] Koppers A.J., Reddy T., O’Bryan M.K. (2011). The role of cysteine-rich secretory proteins in male fertility. Asian J. Androl..

[57] Tadokoro T., Modahl C., Maenaka K., Aoki-Shioi N. (2020). Cysteine-rich secretory proteins (CRISPs) from venomous snakes: An overview of the functional diversity in a large and underappreciated superfamily. Toxins.

[58] Osipov A.V., Levashov M.Y., Tsetlin V.I., Utkin Y.N. (2005). Cobra venom contains a pool of cysteine-rich secretory proteins. Biochem. Biophys. Res. Commun..

[59] Lauridsen L.P., Laustsen A.H., Lomonte B., Gutiérrez J.M. (2017). Exploring the venom of the forest cobra snake: Toxicovenomics and antivenom profiling of *Naja melanoleuca*. J. Proteom..

[60] Nirthanan S., Gwee M.C.E. (2004). Three-finger α-neurotoxins and the nicotinic acetylcholine receptor, forty years on. J. Pharmacol. Sci..

[61] Wang C.I.A., Reeks T., Vetter I., Vergara I., Kovtun O., Lewis R.J., Alewood P.F., Durek T. (2014). Isolation and structural and pharmacological characterization of α-elapitoxin-Dpp2d, an amidated three finger toxin from black mamba venom. Biochemistry.

[62] Pessatti M.L., Fontana J.D., Furtado M.F.D., Guimãraes M.F., Zanette L.R.S., Costa W.T., Baron M. (1995). Screening of Bothrops snake venoms for L-amino acid oxidase activity. Appl. Biochem. Biotechnol..

[63] Arlinghaus F.T., Eble J.A. (2012). C-type lectin-like proteins from snake venoms. Toxicon.

[64] Kini R.M., Evans H.J. (1992). Structural domains in venom proteins: Evidence that metalloproteinases and nonenzymatic platelet aggregation inhibitors (disintegrins) from snake venoms are derived by proteolysis from a common precursor. Toxicon.

[65] Selistre-de-Araujo H.S., Pontes C.L.S., Montenegro C.F., Martin A.C.B.M. (2010). Snake venom disintegrins and cell migration. Toxins.

[66] Župunski V., Kordiš D., Gubenšek F. (2003). Adaptive evolution in the snake venom Kunitz/BPTI protein family. FEBS Lett..

[67] Cardle L., Dufton M.J. (1997). Foci of amino acid residue conservation in the 3D structures of the Kunitz BPTI proteinase inhibitors: How do variants from snake venom differ?. Protein Eng..

[68] Harvey A.L. (2001). Twenty years of dendrotoxins. Toxicon.

[69] Ritonja A., Evans H.J., Machleidt W., Barrett A.J. (1987). Amino acid sequence of a cystatin from venom of the African puff adder (*Bitis arietans*). Biochem. J..

[70] Brillard-Bourdet M., Nguyên V., Ferrer-Di Martino M., Gauthier F., Moreau T. (1998). Purification and characterization of a new cystatin inhibitor from Taiwan cobra (*Naja naja atra*) venom. Biochem. J..

[71] Xie Q., Tang N., Wan R., Qi Y., Lin X., Lin J. (2011). Recombinant snake venom cystatin inhibits the growth, invasion and metastasis of B16F10 cells and MHCC97H cells in vitro and in vivo. Toxicon.

[72] Tan C.H. (2022). Snake Venomics: Fundamentals, Recent Updates, and a Look to the Next Decade. Toxins.

[73] Slagboom J., Kaal C., Arrahman A., Vonk F.J., Somsen G.W., Calvete J.J., Wüster W., Kool J. (2022). Analytical strategies in venomics. Microchem. J..

[74] Li L., Huang J., Lin Y. (2018). Snake venoms in cancer therapy: Past, present and future. Toxins.

[75] Tasoulis T., Pukala T.L., Isbister G.K. (2022). Investigating toxin diversity and abundance in snake venom proteomes. Front. Pharmacol..

[76] Tan K.Y., Wong K.Y., Tan N.H., Tan C.H. (2020). Quantitative proteomics of *Naja annulifera* (sub-Saharan snouted cobra) venom and neutralization activities of two antivenoms in Africa. Int. J. Biol. Macromol..

[77] Laustsen A.H., Lomonte B., Lohse B., Fernández J., Gutiérrez J.M. (2015). Unveiling the nature of black mamba (*Dendroaspis polylepis*) venom through venomics and antivenom immunoprofiling: Identification of key toxin targets for antivenom development. J. Proteom..

[78] Lomonte B., Calvete J.J. (2017). Strategies in “snake venomics” aiming at an integrative view of compositional, functional, and immunological characteristics of venoms. J. Venom. Anim. Toxins Incl. Trop. Dis..

[79] Mouchbahani-Constance S., Sharif-Naeini R. (2021). Proteomic and transcriptomic techniques to decipher the molecular evolution of venoms. Toxins.

[80] Tan C.H., Tan K.Y., Tan N.H. (2019). A proein decomplexation strategy in snake venom proteomics. Funct. Proteom. Methods Protoc..

[81] Wagstaff S.C., Sanz L., Juárez P., Harrison R.A., Calvete J.J. (2009). Combined snake venomics and venom gland transcriptomic analysis of the ocellated carpet viper, *Echis ocellatus*. J. Proteom..

[82] Calvete J.J., Escolano J., Sanz L. (2007). Snake venomics of Bitis species reveals large intragenus venom toxin composition variation: Application to taxonomy of congeneric taxa. J. Proteome Res..

[83] Calvete J.J., Marcinkiewicz C., Sanz L. (2007). Snake venomics of *Bitis gabonica gabonica*. Protein family composition, subunit organization of venom toxins, and characterization of dimeric disintegrins bitisgabonin-1 and bitisgabonin-2. J. Proteome Res..

[84] Dingwoke E.J., Adamude F.A., Mohamed G., Klein A., Salihu A., Abubakar M.S., Sallau A.B. (2021). Venom proteomic analysis of medically important Nigerian viper *Echis ocellatus* and *Bitis arietans* snake species. Biochem. Biophys. Rep..

[85] Juárez P., Wagstaff S.C., Oliver J., Sanz L., Harrison R.A., Calvete J.J. (2006). Molecular cloning of disintegrin-like transcript BA-5A from a *Bitis arietans* venom gland cDNA library: A putative intermediate in the evolution of the long-chain disintegrin bitistatin. J. Mol. Evol..

[86] Ozverel C.S., Damm M., Hempel B.F., Göçmen B., Sroka R., Süssmuth R.D., Nalbantsoy A. (2019). Investigating the cytotoxic effects of the venom proteome of two species of the Viperidae family (*Cerastes cerastes* and *Cryptelytrops purpureomaculatus*) from various habitats. Comp. Biochem. Physiol. Part C Toxicol. Pharmacol..

[87] Bazaa A., Marrakchi N., El Ayeb M., Sanz L., Calvete J.J. (2005). Snake venomics: Comparative analysis of the venom proteomes of the Tunisian snakes *Cerastes cerastes*, *Cerastes vipera* and *Macrovipera lebetina*. Proteomics.

[88] Fahmi L., Makran B., Pla D., Sanz L., Oukkache N., Lkhider M., Harrison R.A., Ghalim N., Calvete J.J. (2012). Venomics and antivenomics profiles of North African *Cerastes cerastes* and *C. vipera* populations reveals a potentially important therapeutic weakness. J. Proteom..

[89] Lauridsen L.P., Laustsen A.H., Lomonte B., Gutiérrez J.M. (2016). Toxicovenomics and antivenom profiling of the Eastern green mamba snake (*Dendroaspis angusticeps*). J. Proteom..

[90] Petras D., Sanz L., Segura Á., Herrera M., Villalta M., Solano D., Vargas M., León G., Warrell D.A., Theakston R.D.G. (2011). Snake venomics of African spitting cobras: Toxin composition and assessment of congeneric cross-reactivity of the Pan-African EchiTAb-Plus-ICP antivenom by antivenomics and neutralization approaches. J. Proteome Res..

[91] Adamude F.A., Dingwoke E.J., Abubakar M.S., Ibrahim S., Mohamed G., Klein A., Sallau A.B. (2021). Proteomic analysis of three medically important Nigerian Naja (*Naja haje*, *Naja katiensis* and *Naja nigricollis*) snake venoms. Toxicon.

[92] Sánchez A., Segura Á., Pla D., Munuera J., Villalta M., Quesada-Bernat S., Chavarría D., Herrera M., Gutiérrez J.M., León G. (2021). Comparative venomics and preclinical efficacy evaluation of a monospecific Hemachatus antivenom towards sub-Saharan Africa cobra venoms. J. Proteom..

[93] Hus K.K., Buczkowicz J., Petrilla V., Petrillová M., Łyskowski A., Legáth J., Bocian A. (2018). First look at the venom of *Naja ashei*. Molecules.

[94] Wong K.Y., Tan K.Y., Tan N.H., Tan C.H. (2021). A neurotoxic snake venom without phospholipase A2: Proteomics and cross-neutralization of the venom from Senegalese cobra, *Naja senegalensis* (Subgenus: Uraeus). Toxins.

[95] Sánchez A., Herrera M., Villalta M., Solano D., Segura Á., Lomonte B., Gutiérrez J.M., León G., Vargas M. (2018). Proteomic and toxinological characterization of the venom of the South African Ringhals cobra *Hemachatus haemachatus*. J. Proteom..

[96] Whiteley G., Casewell N.R., Pla D., Quesada-Bernat S., Logan R.A.E., Bolton F.M.S., Wagstaff S.C., Gutiérrez J.M., Calvete J.J., Harrison R.A. (2019). Defining the pathogenic threat of envenoming by South African shield-nosed and coral snakes (genus Aspidelaps), and revealing the likely efficacy of available antivenom. J. Proteom..

[97] Ainsworth S., Petras D., Engmark M., Süssmuth R.D., Whiteley G., Albulescu L.O., Kazandjian T.D., Wagstaff S.C., Rowley P., Wüster W. (2018). The medical threat of mamba envenoming in sub-Saharan Africa revealed by genus-wide analysis of venom composition, toxicity and antivenomics profiling of available antivenoms. J. Proteom..

[98] Williams D.J., Gutiérrez J.M., Calvete J.J., Wüster W., Ratanabanangkoon K., Paiva O., Brown N.I., Casewell N.R., Harrison R.A., Rowley P.D. (2011). Ending the drought: New strategies for improving the flow of affordable, effective antivenoms in Asia and Africa. J. Proteom..

[99] Habib A.G. (2013). Public health aspects of snakebite care in West Africa: Perspectives from Nigeria. J. Venom. Anim. Toxins Incl. Trop. Dis..

[100] WHO (2017). WHO Expert Committee on Biological Standardization, Sixty-Seventh Report.

[101] Gutiérrez J.M., Vargas M., Segura Á., Herrera M., Villalta M., Solano G., Sánchez A., Herrera C., León G. (2021). *In vitro* tests for assessing the neutralizing ability of snake antivenoms: Toward the 3Rs principles. Front. Immunol..

[102] Gutiérrez J.M., León G., Burnouf T. (2011). Antivenoms for the treatment of snakebite envenomings: The road ahead. Biologicals.

[103] Guidolin F.R., Caricati C.P., Marcelino J.R., da Silva W.D. (2016). Development of equine IgG antivenoms against major snake groups in Mozambique. PLoS Negl. Trop. Dis..

[104] Eursakun S., Simsiriwong P., Ratanabanangkoon K. (2012). Studies on the fractionation of equine antivenom IgG by combinations of ammonium sulfate and caprylic acid. Toxicon.

[105] Simsiriwong P., Eursakun S., Ratanabanangkoon K. (2012). A study on the use of caprylic acid and ammonium sulfate in combination for the fractionation of equine antivenom F(ab’)_2_. Biologicals.

[106] Rojas G., Jiménez J., Gutiérrez J. (1994). Caprylic acid fractionation of hyperimmune horse plasma: Description of a simple procedure for antivenom production. Toxicon.

[107] Moran N.F., Newman W.J., Theakston R.D.G., Warrell D.A., Wilkinson D. (1998). High incidence of early anaphylactoid reaction to SAIMR polyvalent snake antivenom. Trans. R. Soc. Trop. Med. Hyg..

[108] Fernandez S., Hodgson W., Chaisakul J., Kornhauser R., Konstantakopoulos N., Smith A.I., Kuruppu S. (2014). In vitro toxic effects of puff adder (*Bitis arietans*) venom, and their neutralization by antivenom. Toxins.

[109] Segura Á., Villalta M., Herrera M., León G., Harrison R., Durfa N., Nasidi A., Calvete J.J., Theakston R.D.G., Warrell D.A. (2010). Preclinical assessment of the efficacy of a new antivenom (EchiTAb-Plus-ICP^®^) for the treatment of viper envenoming in sub-Saharan Africa. Toxicon.

[110] Gutiérrez J.M., Rojas E., Quesada L., León G., Núñez J., Laing G.D., Sasa M., Renjifo J.M., Nasidi A., Warrell D.A. (2005). Pan-African polyspecific antivenom produced by caprylic acid purification of horse IgG: An alternative to the antivenom crisis in Africa. Trans. R. Soc. Trop. Med. Hyg..

[111] Calvete J.J., Cid P., Sanz L., Segura Á., Villalta M., Herrera M., León G., Harrison R., Durfa N., Nasidi A. (2010). Antivenomic assessment of the immunological reactivity of EchiTAb-Plus-ICP, an antivenom for the treatment of snakebite envenoming in sub-Saharan Africa. Am. J. Trop. Med. Hyg..

[112] Sánchez A., Segura Á., Vargas M., Herrera M., Villalta M., Estrada R., Wu F., Litschka-Koen T., Perry M.A., Alape-Girón A. (2017). Expanding the neutralization scope of the EchiTAb-plus-ICP antivenom to include venoms of elapids from Southern Africa. Toxicon.

[113] Djameh G.I., Nyarko S., Tetteh-Tsifoanya M., Marfo F.M., Adjei S., Blay E.A., Anang A.K., Ayi I. (2021). Preclinical immuno-recognition and neutralization of lethality assessment of a new polyvalent antivenom, VINS snake venom antiserum—African IHS^®^, against envenomation of ten African viperid and elapid snakes. J. Sci. Res. Rep..

[114] Makran B., Fahmi L., Pla D., Sanz L., Oukkache N., Lkhider M., Ghalim N., Calvete J.J. (2012). Snake venomics of *Macrovipera mauritanica* from Morocco, and assessment of the para-specific immunoreactivity of an experimental monospecific and a commercial antivenoms. J. Proteom..

[115] Sánchez L.V., Pla D., Herrera M., Chippaux J.P., Calvete J.J., Gutiérrez J.M. (2015). Evaluation of the preclinical efficacy of four antivenoms, distributed in sub-Saharan Africa, to neutralize the venom of the carpet viper, *Echis ocellatus*, from Mali, Cameroon, and Nigeria. Toxicon.

[116] Potet J., Smith J., McIver L. (2019). Reviewing evidence of the clinical effectiveness of commercially available antivenoms in sub-saharan Africa identifies the need for a multi-centre, multi-antivenom clinical trial. PLoS Negl. Trop. Dis..

[117] Harrison R.A., Oluoch G.O., Ainsworth S., Alsolaiss J., Bolton F., Arias A.S., Gutiérrez J.M., Rowley P., Kalya S., Ozwara H. (2017). Preclinical antivenom-efficacy testing reveals potentially disturbing deficiencies of snakebite treatment capability in East Africa. PLoS Negl. Trop. Dis..

[118] Méndez I., Gutiérrez J.M., Angulo Y., Calvete J.J., Lomonte B. (2011). Comparative study of the cytolytic activity of snake venoms from African spitting cobras (*Naja* spp., Elapidae) and its neutralization by a polyspecific antivenom. Toxicon.

[119] Alangode A., Rajan K., Nair B.G. (2020). Snake antivenom: Challenges and alternate approaches. Biochem. Pharmacol..

[120] Harrison R.A. (2004). Development of venom toxin-specific antibodies by DNA immunisation: Rationale and strategies to improve therapy of viper envenoming. Vaccine.

[121] Wagstaff S.C., Laing G.D., Theakston R.D.G., Papaspyridis C., Harrison R.A. (2006). Bioinformatics and multiepitope DNA immunization to design rational snake antivenom. PLoS Med..

[122] Laustsen A.H., María Gutiérrez J., Knudsen C., Johansen K.H., Bermúdez-Méndez E., Cerni F.A., Jürgensen J.A., Ledsgaard L., Martos-Esteban A., Øhlenschlæger M. (2018). Pros and cons of different therapeutic antibody formats for recombinant antivenom development. Toxicon.

[123] Lipps B.V. (1999). Anti-lethal factor from opossum serum is a potent antidote for animal, plant and bacterial Toxins. J. Venom. Anim. Toxins.

[124] Komives C.F., Sanchez E.E., Rathore A.S., White B., Balderrama M., Suntravat M., Cifelli A., Joshi V. (2017). Opossum peptide that can neutralize rattlesnake venom is expressed in *Escherichia coli*. Biotechnol. Prog..

[125] Albulescu L.O., Xie C., Ainsworth S., Alsolaiss J., Crittenden E., Dawson C.A., Softley R., Bartlett K.E., Harrison R.A., Kool J. (2020). A therapeutic combination of two small molecule toxin inhibitors provides broad preclinical efficacy against viper snakebite. Nat. Commun..

